# The Role of CT Imaging in a Fractured Coronary Stent with Pseudoaneurysm Formation

**DOI:** 10.3390/diagnostics14080840

**Published:** 2024-04-18

**Authors:** Radu Octavian Baz, George Gherghescu, Adnan Mustafa, Mihaly Enyedi, Cristian Scheau, Radu Andrei Baz

**Affiliations:** 1 Clinical Laboratory of Radiology and Medical Imaging, “Sf. Apostol Andrei” County Emergency Hospital, 900591 Constanta, Romania; andreibaz@yahoo.com (R.O.B.); gherghescu.g@gmail.com (G.G.);; 2 Department of Radiology and Medical Imaging, Faculty of Medicine, “Ovidius” University, 900527 Constanta, Romania; 3 Department of Cardiology, “Sf. Apostol Andrei” County Emergency Hospital, 900591 Constanta, Romania; drmustafa.adnan@yahoo.com; 4 Department of Anatomy, The “Carol Davila” University of Medicine and Pharmacy, 050474 Bucharest, Romania; mihaly.enyedi@umfcd.ro; 5 Department of Radiology, “Victor Babes” Center for Diagnosis and Treatment, 030303 Bucharest, Romania; 6 Department of Physiology, The “Carol Davila” University of Medicine and Pharmacy, 050474 Bucharest, Romania; 7 Department of Radiology and Medical Imaging, “Foisor” Clinical Hospital of Orthopaedics, Traumatology and Osteoarticular TB, 030167 Bucharest, Romania

**Keywords:** computed tomography, medical imaging, angiography, stent fracture, pericardial hematoma, coronary pseudoaneurysm

## Abstract

We report a case of a 63-year-old male patient with multiple cardiovascular risk factors and previous myocardial infarction who was referred to the emergency department on September 2023 with symptoms and clinical and biological data consistent with an acute coronary event. A coronary angiography revealed severe ostial stenosis of the left anterior descending artery (LAD) and intrastent thrombotic occlusion in the first two segments of the LAD. Two drug-eluting stents were implanted and the patient was discharged when hemodynamically stable; however, three weeks later, he returned to the emergency department complaining of fever, anterior chest pain, dyspnea at rest, and high blood pressure values at home. High levels of troponin T, C-reactive protein, and NT-proBNP were detected and blood cultures showed methicillin-resistant Staphylococcus aureus. The computed tomography (CT) examination showed a saccular dilatation had developed between two fragments of a stent mounted at the level of the LAD, surrounded by a hematic pericardial accumulation. LAD pseudoaneurysm ablation and a double aortocoronary bypass with inverted saphenous vein autograft were performed and the patient showed a favorable postoperative evolution. In this case, surgical revascularization was proven to be the appropriate treatment strategy, demonstrating the need to choose an individualized therapeutic option depending on case-specific factors.

**Figure 1:**
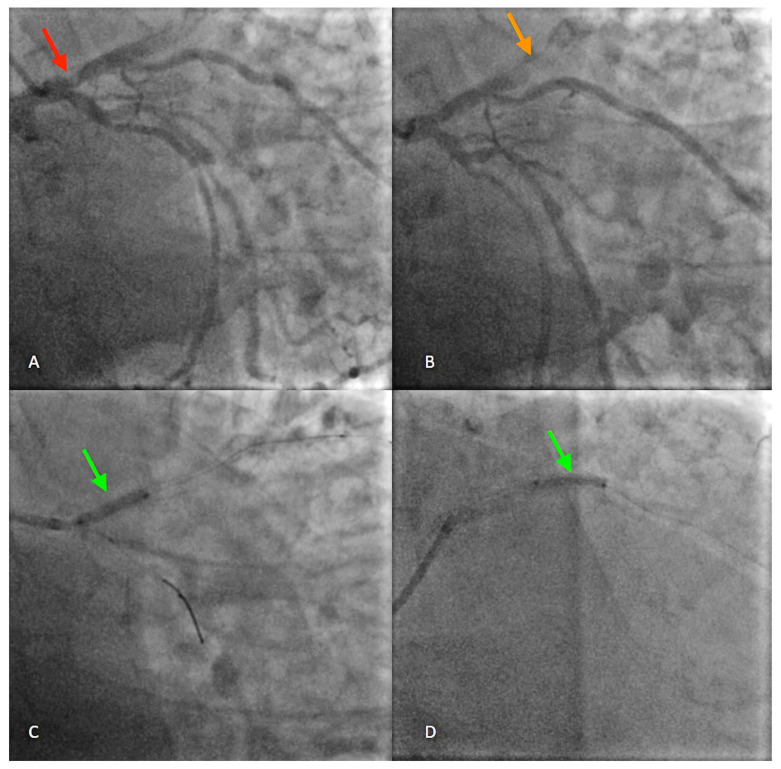
A 63-year-old male patient, with dyslipidemia, hypertension, diabetes mellitus type 2, history of smoking, severe coronary disease, and prior acute myocardial infarction was referred to the emergency department with constrictive chest pain and sweating for 6 h. The patient had a history of coronary stent implantation in the right and anterior descending coronary arteries 6 years prior, and, upon initial examination, had blood pressure (BP) in both arms of 120/60 mm Hg, heart rate (HR) of 90 bpm, regular rhythm, normal heart sounds on auscultation, no dyspnea or signs of systemic or pulmonary congestion. Blood tests revealed increased levels of markers of cardiac necrosis, mild anemia, hyperglycemia, hyperuricemia, hyponatremia, and acidosis. ECG showed ST-segment elevations of up to 5 mm in leads V2–V6, Q waves in leads V1–V4, and bifascicular block with complete right bundle branch block and left anterior fascicular block. TTE reveals dyskinesia of the affected myocardial territory, namely the interventricular septum and the anterolateral wall in the apical two-thirds, respectively, systolic dysfunction, with an ejection fraction of 30%. Coronary angiography revealed severe ostial stenosis of the LAD ((**A**), red arrow) and intrastent thrombotic occlusion in the first two segments of the LAD ((**B**), orange arrow), which is why pharmacologically active stents XIENCE PRO 4.0/23 mm, BIOMIME 2.5/13 mm, and BIOMIME 4.0/13 mm were implanted in the proximal and mid parts of the LAD, each stent extending beyond the previous one, with the restoration of blood flow ((**C**,**D**), green arrows).

**Figure 2:**
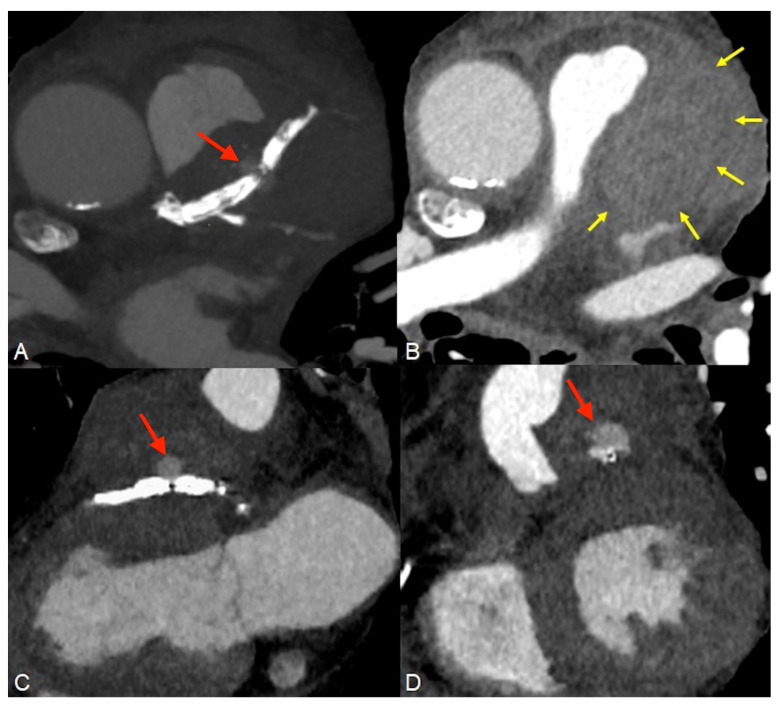
Approximately three weeks later, the patient returned to the emergency department complaining of fever, anterior chest pain, dyspnea at rest, and high BP values at home. ECG findings were similar to the previous exam. Laboratory analyses again showed increased values of troponin T (256.4 pg/mL), C-reactive protein (8 mg/dL), and NT-proBNP (11,594 Pg/mL). Peripheral blood cultures and pericardial fluid analysis were performed, and the culture media developed colonies of facultatively anaerobic Staphylococcus aureus, resistant to methicillin. A contrast-enhanced chest computed tomography (CT, GE Revolution 256 × 2) examination was ordered which did not detect acute lung lesions. However, complete separation of the proximal LAD stent into two fragments, with a distance of ~5 mm in between, was observed ((**A**), red arrow). A pericardial hematoma around the trunk of the pulmonary artery ((**B**), yellow arrows) and a saccular enlargement with transverse diameters of ~10/12 mm between the two stent fragments were identified ((**C**,**D**), red arrows). Therefore, the CT diagnosis was a fracture of the distal segment of the proximal LAD stent associated with pseudoaneurysm of the anterior descending artery and pericardial hematoma. Stent fracture can occur due to various causes including heavy calcification, left ventricle remodeling, stent length and overlap, arterial flexion, implant duration, and material fatigue; ongoing efforts aim to increase lifespans by improving flexibility and tear resistance [1,2,3].

**Figure 3:**
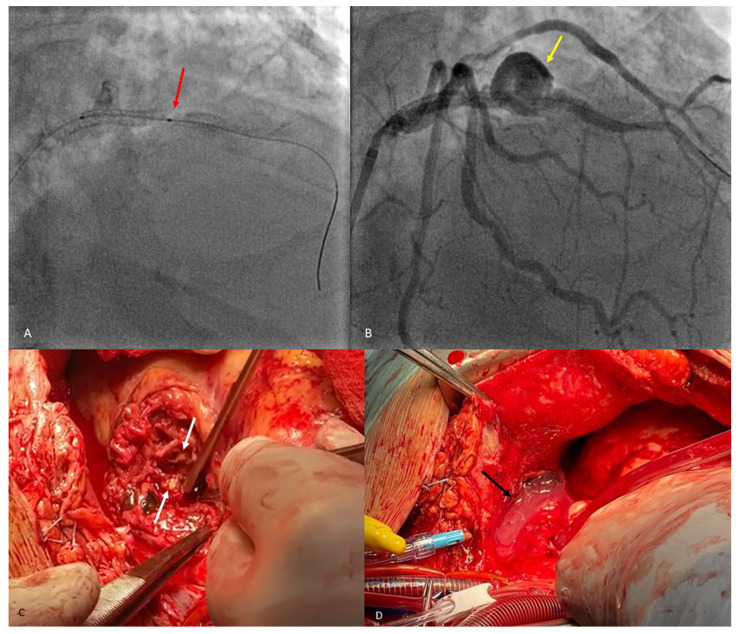
A classic coronary angiography was performed next, where a type IV distal stent fracture involving the proximal LAD stent with dehiscence and slight migration ((**A**), red arrow) associated with a coronary pseudoaneurysm ((**B**), yellow arrow) were observed. An attempt was made to install a pharmacologically active post-dilation balloon stent in order to treat the pseudoaneurysm, but without success, which is why the patient was transferred to the cardiovascular surgery ward. The surgical procedure was performed under general anesthesia with orotracheal intubation and extracorporeal circulation, and the final diagnosis was a stent fracture associated with pseudoaneurysm formation and infected pericardial hematoma. Intraoperative view of the complete stent fracture and separation ((**C**), white arrows). Infected pericardial hematoma ((**D**), black arrow). The case is atypical, as the stent fracture was type IV (complete transverse fracture of the stent with separation into two fragments and displacement) and occurred at the level of the anterior descending artery. The most common stent fractures reported in the literature involve the right coronary artery, with a more tortuous course, and type III and IV fractures have the lowest incidences [4]. Coronary pseudoaneurysms following stenting are rare complications with an incidence of 0.3–6% and usually arise approximately 6–9 months after the intervention, but cases have been reported earlier than 2 months after the procedure [5]. Unlike similar reports in the literature [6], the attempt to implant a new drug-eluting stent in our patient failed. Finally, surgical revascularization proved to be the appropriate treatment strategy. After removing the pseudoaneurysm and performing an aorto-coronary bypass, the patient showed a favorable recovery and was discharged in a stable condition. Invasive coronary angiography is considered the gold standard for diagnosing intra-stent restenosis, although the invasive nature of the procedure comes with associated risks of mortality and morbidity. Computed tomography angiography (CTA) is a non-invasive imaging technique highly beneficial for follow-up consultations. Coronary artery intrastent restenosis detection has been determined to have high specificity when at least a 64-multislice CT technique is used. CTA can and should also be used when varying symptoms appear after an interventional coronary procedure [7].

## Data Availability

The data that support the findings of this study are not openly available due to reasons of sensitivity and are available from the corresponding author upon reasonable request.
